# A Golden Age for Working with Public Proteomics Data

**DOI:** 10.1016/j.tibs.2017.01.001

**Published:** 2017-05

**Authors:** Lennart Martens, Juan Antonio Vizcaíno

**Affiliations:** 1 Medical Biotechnology Center, VIB, Ghent, Belgium; 2 Department of Biochemistry, Ghent University, Ghent, Belgium; 3 Bioinformatics Institute Ghent, Ghent University, Ghent, Belgium; 4 European Molecular Biology Laboratory, European Bioinformatics Institute (EMBL-EBI), Wellcome Trust Genome Campus, Hinxton, Cambridge CB10 1SD, UK

## Abstract

Data sharing in mass spectrometry (MS)-based proteomics is becoming a common scientific practice, as is now common in the case of other, more mature ‘omics’ disciplines like genomics and transcriptomics. We want to highlight that this situation, unprecedented in the field, opens a plethora of opportunities for data scientists. First, we explain in some detail some of the work already achieved, such as systematic reanalysis efforts. We also explain existing applications of public proteomics data, such as proteogenomics and the creation of spectral libraries and spectral archives. Finally, we discuss the main existing challenges and mention the first attempts to combine public proteomics data with other types of omics data sets.

## Trends

The field of proteomics has matured and diversified substantially over the past 10 years.

Proteomics data are increasingly shared through centralized, public repositories.

Standardization efforts have ensured that a large proportion of these public data can be read and processed by any interested researcher.

Because any proteomics data set is only partially understood, there is great opportunity for (orthogonal) reuse of public data.

While public proteomics data has so far remained outside ethics and privacy discussions, recent work indicates that there is an inherent risk.

## MS-Based Proteomics Data in the Public Domain

MS-based proteomics approaches have evolved rapidly over recent years. These approaches are therefore increasingly used to disentangle intricate biological questions, often together with other omics disciplines (e.g., genomics, transcriptomics, metabolomics) 1, 2, 3. A key signal of the maturity of the field is the common acceptance of public data sharing (as embraced earlier in genomics and transcriptomics) as good scientific practice. This important change of mentality has been triggered by requirements from scientific journals and funding agencies on the one hand [4] and by the availability of reliable and more user-friendly resources and tools to support data sharing on the other hand 5, 6.

The first MS proteomics resources were set up more than 10 years ago, notably PeptideAtlas [7], GPMDB [8], and PRIDE (now renamed PRIDE Archive) 9, 10, and these continue to be leading resources worldwide. Through the years, other proteomics resources have appeared and, regrettably, also disappeared [11]. However, at present the field is experiencing a ‘golden age’ for MS proteomics resources. Several notable resources have come into being, including MassIVE (http://massive.ucsd.edu/), jPOST (http://jpost.org/), the Human Proteome Map (http://www.humanproteomemap.org/), ProteomicsDB (https://www.proteomicsdb.org/), and Chorus (https://chorusproject.org/) (for a recent review, see [12]). In 2011 some of the most prominent resources in the field came together and started to collaborate formally, resulting in unified submission and data dissemination practices within the ProteomeXchange (PX) Consortium [13] (http://www.proteomexchange.org/). At present, the PX members are PRIDE, PeptideAtlas (including the PASSEL resource for targeted proteomics data [14]), MassIVE, and jPOST.

Most of the data sets publicly available correspond to human and the main model organisms. However, non-model organisms are also increasingly well represented. Data sets from more than 900 different taxonomic identifiers are available in the various PX repositories [15]. The data submission process has been described in detail elsewhere 12, 16.

Because of these developments, we believe that the field is now filled with opportunities for those wanting to extract new knowledge from this abundance of data. While new in proteomics, such orthogonal reprocessing of public data is already common in even more mature fields [17]. With a few notable exceptions (e.g., 7, 8, 18, 19, 20), the data so far remain largely untouched.

In this Opinion article, we discuss some of the challenges and possible future directions for proteomics data resources and convince researchers of the utility of making their data publicly available. Finally, we demonstrate the high number of exciting possibilities available for scientists willing to work with these data.

## Overview of the Ways in which Proteomics Data Can Be Reused

**Figure 1:**
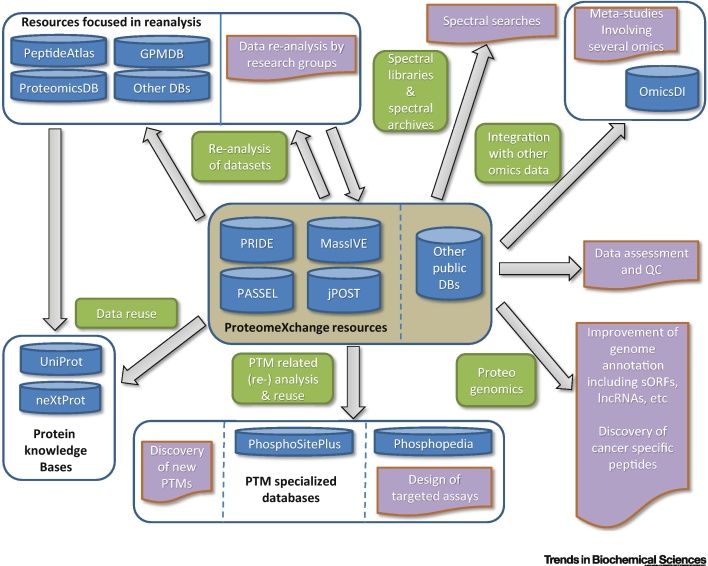
Key Figure: Overview of the Main Uses and Applications of Public Proteomics Data Sets

In proteomics the number of data types and their corresponding data formats can be overwhelming. The main data types that need to be stored by proteomics repositories are raw (MS data generated by the mass spectrometer) and analyzed (for identification and quantification-based analyses). For PX data sets, it is mandatory to provide both data types, since they provide complementary information and enable different types of data reuse. The availability of raw data enables a full reanalysis of the data sets while the analyzed data can be used, among other things, for visualizing and assessing the results reported in a given study. The development of data standards has contributed to simplifying the use of public proteomics data for scientists (Box 1). In a recent review [21], together with other colleagues we established four categories of public proteomics data use: (i) use; (ii) reuse; (iii) reprocess; and (iv) repurpose. An overview of the main applications is provided in Figure 1 (Key Figure).

A simple example of the direct use of data is given by connecting information between the above-cited proteomics data resources and protein knowledge bases, such as UniProt [22] and neXtProt [23]. This type of use is quite impactful because such knowledge bases are the most likely conduit through which researchers in the broader life sciences will benefit from these data.

In the case of reuse, public data are not only connected with complementary knowledge but also reused in novel experiments with the potential to generate new knowledge. The creation and use of spectral libraries and spectral archives represent a clear example (Box 2). In addition, one generic type of data reuse, also popular in other disciplines, is the analysis of data from a large number of independent data sets in combination, a so-called meta-analysis study, to extract new knowledge not accessible from any one individual data set. Although there are some notable examples of this type of study in the field (e.g., 24, 25), such reuse remains relatively scarce [21]. This comparative lack of published studies belies the many opportunities that are available through such endeavors, however.

In the case of reprocess, public data are reanalyzed to provide an updated view on the results as protein sequence databases (used by the majority of search engines in proteomics) evolve and become more accurate. Such analyses, which are also common in other disciplines, have goals the same as or similar to the original experiment, although the reprocess can deliver novel findings. Resources such as PeptideAtlas and GPMDB routinely reprocess many datasets using their dedicated bioinformatics tools and pipelines. The results from PeptideAtlas are organized into builds, each including data from a single species proteome (e.g., human, pig, *Candida albicans*) or subproteome (e.g., human plasma). Each build is generated by reanalyzing the raw MS/MS spectra compiled by PeptideAtlas over the years or from data from other public repositories, especially PRIDE. Analogously, GPMDB reprocesses MS/MS data provided by users or raw data stored in other repositories. Both PeptideAtlas and GPMDB are actively contributing to the Human Proteome Project (HPP) and are providing guidelines and a consensus up-to-date list (updated each year) of the human proteins that have been detected by MS [26]. In the context of the HPP, both resources are working closely together with neXtProt in the process providing a nice example of a reprocess effort by proteomics data resources that leads to use of the obtained results by a knowledge base.

Finally, repurposing includes all those cases where the data are considered anew in a context that differs from the original experiment. Two attractive applications of this type of study are proteogenomics approaches and the discovery of novel post-translational modifications (PTMs). Of course, before repurposing any data set it is important to obtain an idea about its suitability to the purpose at hand. This is typically accomplished through appropriate types of quality control (QC). In the following sections, we therefore first discuss QC of (public) proteomics data and then move on to the proteogenomics and PTM use cases.

## QC of Proteomics Data

In any analytical discipline, QC is very important [27]. However, QC has historically not been as well developed in proteomics as in, for instance, small-molecules MS. Here, too, public data availability can play a role as it enables *a posteriori* QC of the data [28]. Ideally, all data in repositories should be linked to objective quality metrics, but this process has barely started 5, 29 as appropriate software tools have only recently become available 30, 31. At present, proteomics resources are assessing the internal consistency of the data submitted (e.g., correspondence between the mass spectra and identification results), detecting clear annotation errors (e.g., related to PTMs), and ensuring an acceptable level of technical and biological metadata. In addition, as a key point, the availability of free-to-use tools such as PRIDE Inspector 5, 32 enables potential errors to be detected by anyone in the community.

Of course, QC metric calculation at the level of proteomics resources can serve only as a postmortem, as potential issues can no longer be solved at that point. A perfect situation would therefore see QC metrics produced in parallel with data acquisition in the laboratory and subsequently communicated to repositories alongside the data.

## Proteogenomics

In proteogenomics proteomics data is combined with genomics and/or transcriptomics information, typically using sequence databases generated from DNA sequencing efforts, RNA-seq experiments, or Ribo-seq approaches, among others. The promise of these approaches is that, if peptides are detected that cover events such as novel splice junctions, long noncoding RNAs (lncRNAs), small open reading frames (ORFs), or pseudogenes, genome annotation can be improved [33].

Proteogenomics approaches benefit greatly from the availability of public data sets because it is likely that the number of novel events detected in a single data set will be very small compared with the data acquisition effort. Especially in the case of well-studied organisms (e.g., human), a large number of public data sets are, however, already available. Several studies have been published where public data have been used in proteogenomics projects for human (e.g., 19, 34), mouse [35], and rat [36], among other organisms. In addition, the complete compendium of public data for humans has been reanalyzed to provide evidence-of-existence annotation for the human lncRNAs stored in LNCipedia [37]. The latest trend is to use public data together with Ribo-seq data for the determination of small ORFs [38].

In our opinion one of the key issues in proteogenomics at present is the lack of connection between the researchers who performed the analyses and the resources that can update genome annotation based on these new findings. Thankfully, this situation is improving, as common genomics data formats are currently being extended by the Human Proteome Organization (HUPO) Proteomics Standards Initiative (PSI) to also support proteomics information [e.g., the proBed (http://www.psidev.info/probed) and proBAM (http://www.psidev.info/probam) data formats]. These standard formats can already be used to generate ‘Track Hubs’ [39], which can be provided by any interested third parties and which can be automatically integrated into genome browsers such as Ensembl and the UCSC Genome Browser. While this mechanism is not yet fully mature, the coming months are likely to see substantial improvements.

The other big issue in proteogenomics studies is the accumulation of false positives, as exemplified in the human proteome draft papers mentioned in Box 3. Much more restrictive quality criteria should be established for peptides describing novel genomics events [33]. Moreover, the enlarged sequence search space of typical proteogenomics searches can lead to undesirable ambiguity of identification [40].

## PTM-Related Studies

Proteomics approaches (both MS and antibody based) provide the sole means of detecting and localizing protein PTMs. Of the many known PTMs, phosphorylation is by far the most studied, and as a result the number of phosphoproteomics data sets in the public domain is large and growing. Several highly valuable resources, such as PhosphoSitePlus [41], are specialized in compiling phosphorylation-related information from various sources, including MS proteomics resources, constituting another elegant example of a simple use of the data.

However, public datasets are also being reanalyzed to extract new knowledge in the context of PTM-related research. For instance, the spread of detected phosphosites on protein structure has been analyzed, and in a recent study three large phosphoproteomics data sets (including two public ones) were reanalyzed as a starting point for the generation of robust targeted MS assays [20]. The resulting assay data are available in a novel resource called Phosphopedia. The same approach could be applied to other PTMs as the number of relevant public data sets grows. Finally, as mentioned above, it is also possible to repurpose the analysis of existing data sets to look for PTMs that were not initially considered in the searches. To our knowledge the only successful studies so far have used enriched phosphoproteomics data sets to find serendipitously co-enriched peptides bearing unusual modifications 42, 43.

Glycosylation represents a widely occurring PTM. It would be highly beneficial to achieve a closer interaction between existing proteomics and glycomics resources [44]. At present, to the best of our knowledge, these efforts have barely started.

## Integration of Proteomics Data Sets with Other Public Omics Data Sets

It becomes steadily easier as well as more rewarding to combine public proteomics data with other public omics data, which opens a multitude of novel opportunities for data scientists.

Proteogenomics approaches have recently, for instance, been used to study various cancers, focusing on cancer-specific peptides for diagnostic and therapeutic purposes. The Clinical Proteomic Tumor Analysis Consortium (CPTAC) of the National Cancer Institute (NCI) has released several high-profile studies for several tumor types, including colorectal [45], breast [46], and ovarian [47]. These data are all publicly available at least through the CPTAC Data Portal and represent a typical example where the protein sequence databases used for the analysis are directly derived from the corresponding exome sequences from the cancer samples.

However, at present, in most cases it is not trivial for researchers to connect data sets that have been generated in multiomics studies. Two exceptions are consortia that have their own data repository or portal (like CPTAC) and organism-specific resources such as the ‘*Saccharomyces* Genome database’. This is because the first substantial obstacle for integrative data scientists is finding suitable data sets to link. This key issue is being addressed by the Omics Discovery Index (OmicsDI) (http://www.omicsdi.org/), a recently released portal for the discovery and access of data sets from various omics approaches and online resources [48]. Among other features, OmicsDI represents the concept of multiomics data sets by connecting different omics datasets cited in the same publication. For instance, in September 2016 OmicsDI knew about more than 30 multiomics data sets that contain both proteomics data and the corresponding gene expression data. Indeed, the first examples of studies combining public proteomics and gene expression data sets already exist [49]. This type of multiomics study involving proteomics data will only grow as public data deposition generalizes for all omics disciplines and the data sets are better connected. Publications combining proteomics and lipidomics/metabolomics approaches are starting to appear [50].

## Challenges

The lack of experimental and technical metadata has been highlighted many times as the main issue for the reuse of biological data, and particularly in proteomics [51]. The metadata requirements of proteomics resources in general are much less comprehensive than those of equivalent resources from more mature omics fields, leading to more pronounced annotation problems for proteomics data. In our experience there needs to be a balance between the required amount of metadata and the willingness of researchers to share their data. Scientists try to avoid ‘administrative’ work as much as possible. Because the data-sharing culture started more recently in proteomics than in other disciplines, the main focus so far has been facilitating the process of data sharing as much as possible, from both a technical and a time-efficiency point of view. Raising the bar in terms of metadata requirements is an achievable goal, as far as proteomics resources have the means to evolve their systems and tools. Unfortunately, the latter can be challenging in the current funding situation as it is often perceived that all issues in this area have been solved. In this context, as a ‘silver lining’, it is important to highlight that the increased adoption of the data standards is key to improving the situation, as much metadata (especially the proteomics-specific metadata) can be extracted automatically from the acquired data files instead of having to be entered manually by the submitters.

In the near future, one challenge that may arise is the existence of limited access to human clinical proteomics data, as is common today for genomics and transcriptomics data sets, where specialized, access-controlled resources such as the European Genotype Archive (EGA) (https://www.ebi.ac.uk/ega/) and dbGaP (http://www.ncbi.nlm.nih.gov/gap/) have to be used. Access to these data is granted only after applications are reviewed by an ethics committee. The first studies describing the possibility of recognizing specific patients using proteomics data have just been published 52, 53. Whether access limitations will ultimately apply to clinical proteomics data remains to be seen, but undoubtedly this topic will become an important matter for discussion in the near future.

## Concluding Remarks and Future Perspectives

We hope to have convinced the reader that there is a bright future for data scientists in the MS proteomics field (see Outstanding Questions). Regrettably, the term ‘research parasites’ has recently been bandied about to describe those who work with publicly available data generated by others [54]. In our opinion this term is not justified for two reasons. First, the scientists who generated the data originally should, of course, be acknowledged and given proper recognition and citation, and in our experience this has been, and remains, the default scientific behavior. Nevertheless, there will always be researchers who fail to cite their sources adequately and this is certainly not unique to the reuse of public data. However, public data sharing should not be stalled because of a small minority of researchers that are not complying with these basic practices. Second, if data has not yet been analyzed in full by the originator at the time of publication, it can hardly be termed parasitism if others attempt to further optimize the value of these data by analyzing them, especially if they do so in innovative and orthogonal ways. Instead, one would expect that any mature field of research should welcome novel insights that can be derived from their existing data. Perhaps the most compelling argument of all is that, in the end, most of the research is funded by public money, so to make the data freely available, at least after publication, maximizes the value of the funds provided.

Outstanding Questions

• What (novel) information can still be obtained from the roughly two-thirds of unidentified fragmentation spectra that are typically acquired in a proteomics experiment?
• Linked to the previous question: can we develop sensitive as well as specific identification algorithms for proteomics data that no longer need to rely on very narrowly defined candidate analytes, as, for instance, obtained from a protein sequence database?
• Can we extract information from the combined (human) proteome data in public data repositories to guide us to the most promising tissue types and sample protocols to obtain more complete coverage of the (human) proteome?
• How can we make the most of the combined public omics data from different fields and what, if any, additional infrastructure needs to be put in place to allow these data to become discoverable and to allow these data to be easily connected?
• How can we accelerate the as-yet very limited use of proteomics data as a means of enhancing current genome annotation efforts?
• What privacy and ethics issues will proteomics data raise in the future and what can the field do to adequately prepare for these?
• How can we increase the use of spectral libraries generated from public proteomics data in existing analysis workflows?
• How can the lingering notion that reuse of public data in the life sciences is equivalent to theft or parasitism be overturned?

## Box 1 Data Standards in Proteomics

The availability of data standards is crucial to public data sharing and such standards are therefore instrumental in the advancement of any research field. For the field of MS-based proteomics, the PSI of the HUPO has taken the leading role in developing the required controlled vocabularies, standard file formats, and minimal reporting requirements for various aspects of MS proteomics data (for a recent review, see [55]). The most popular formats are: (i) mzML, which stores raw MS data as well as processed peak list spectra [56]; (ii) mzIdentML, which captures the peptide and protein identifications derived from MS data [57]; and (iii) mzQuantML, which captures detailed quantitative information [58]. Besides these three XML-based formats, a simpler, tab-delimited format called mzTab has been released that stores both identification and quantification information [59].

Whereas mzML is already well adopted and mzIdentML is on track to become a successful format, the adoption of data standards for quantitative data has not yet taken off. This is mainly due to the lack of support for these more recent standards by most popular tools. A recent effort to create a standard to express QC metrics, named qcML, has also been proposed [60]. Because standardization is so crucial to efficient and accurate data sharing, it is vital that all of these relevant standards become widely adopted as soon as possible. This will in turn enable proteomics data to become fully accessible and reusable and will foster innovative approaches to make the most of these data. Disciplines such as genomics and transcriptomics provide excellent examples for the type of rapid development that can occur when standards adoption becomes ubiquitous.

Alt-text: Box 1

## Box 2 Spectral Libraries and Spectral Archives

In addition to the widely used sequence database-based approaches, several alternative analysis techniques exist that can potentially improve the rate of identified spectra. One of the most popular approaches is the use of spectral library searches, which rely on collections of previously identified experimental spectra (or consensus versions of those) called spectral libraries. The creation of such libraries benefits enormously from the accumulation of data in the public domain, which allows the libraries to expand in terms of different species, mass spectrometers, and experimental approaches. PeptideAtlas, GPMDB, PRIDE, and other organizations such as the National Institute of Standards and Technology (NIST) all provide such spectral libraries. A recent use of spectral libraries is in the analysis of Data Independent Acquisition (DIA) data, such as SWATH-MS [61]. For this particular use case, spectral libraries are typically generated by individual laboratories using their own data. However, a new tool has been recently developed that can make use of spectral libraries generated elsewhere, even in other types of mass spectrometers [62]. However, it should be noted that reliable and consistent retention times are essential for this approach.

The main issues for spectral libraries are the accumulation of false-positive identifications that may be present in the original spectral libraries and the difficulty of finding novel peptides not previously observed. This last limitation hinders spectral library-based analysis approaches in SWATH-MS where, as mentioned, individual groups need to generate comprehensive spectral libraries that need to cover all of the different samples and scenarios considered in the study. This can represent a major effort that should not be underestimated.

Spectral archives are an extension of spectral libraries comprising collections of mass spectra that can include unidentified spectra rather than only previously identified spectra. PRIDE has recently provided the first spectral archive version of a proteomics repository [63]. This archive was created using a spectrum-clustering approach that enabled the detection of millions of consistently observed but unidentified spectra across hundreds of public data sets. By using alternative analysis approaches, the authors managed to identify around 20% of these spectra, which illustrates that more efforts are still needed to identify the remaining ones. It is not unreasonable to assume that a proportion of these unidentified spectra correspond to unknown peptide sequence variants or unexpected PTMs.

Alt-text: Box 2

## Box 3 Human Proteome Drafts and Discussions

One of the benefits of public data availability is that it enables independent assessment of the results described in scientific publications. In the proteomics field, too, there have been several examples of widely debated, controversial findings [21]. These healthy discussions were triggered and enabled naturally by the public availability of the corresponding data sets. The most prominent example occurred recently, surrounding the two human proteome drafts published by the Pandey [64] and Kuster [18] groups. To their considerable credit, both teams made their corresponding raw MS data publicly available and, moreover, created online resources to facilitate access to their results: the Human Proteome Map and ProteomicsDB, respectively. Based on thus-enabled detailed scrutiny, the findings in these two papers have been criticized by many, mainly due to a lack of stringency in data analysis (e.g., in [65]). This causes the accumulation of false-positive identifications due to associated underestimation of the false-discovery rate (FDR). Meanwhile, several groups have been extracting novel conclusions from reanalysis of these data [19], and it is expected that these extensive data sets will continue to generate novel findings for some time. As an important aside, it is particularly interesting to note that the human proteome draft generated by the Kuster group already relies on reanalyses of other researchers’ data sets from public resources for around 60% of its contents.

Additionally, data reliability in the field can also be improved indirectly by facilitating the development and benchmarking of improved software analysis tools, which will potentially increase the data reliability of future studies. In this context of software development, there are multiple examples of reuse of public data sets [21]. The existence of benchmarking data sets is essential and efforts have been made to specifically promote their public availability [66].

Alt-text: Box 3
